# Video-assisted thoracoscopic surgery for recurrent pneumothorax in pulmonary lymphangioleimyomatosis with tuberous sclerosis complex

**DOI:** 10.1186/1749-8090-8-101

**Published:** 2013-04-18

**Authors:** Chia-Fen Tsai, Chen-Hao Hsiao, Jang-Ming Lee, Ke-Cheng Chen, Ming-Jium Shieh, Hong-Shiee Lai, Jin-Shing Chen

**Affiliations:** 1 Institute of Biomedical Engineering, College of Medicine and College of Engineering, National Taiwan University, Taipei, Taiwan; 2 Department of Surgery, National Taiwan University Hospital Yun-Lin Branch, Yun-Lin County, Taiwan; 3 National Taiwan University Hospital Chu-Tung Branch, Hsin-Chu County, Taiwan; 4 National Taiwan University Hospital Hsin-Chu Branch, Hsin-Chu, Taiwan; 5 Department of Dermatology, National Taiwan University Hospital and National Taiwan University College of Medicine, Taipei, Taiwan; 6 Department of Surgery, National Taiwan University Hospital and National Taiwan University College of Medicine, Taipei, Taiwan; 7 Department of Surgery, Cheng Hsin General Hospital, Taipei, Taiwan; 8 Genome and Systems Biology Degree Program, National Taiwan University and Academia Sinica, Taipei, Taiwan

**Keywords:** Pneumothorax, Pulmonary lymphangioleiomyomatosis, Tuberous sclerosis complex, Video-assisted thoracoscopic surgery

## Abstract

Pneumothorax in pulmonary lymphangioleiomyomatosis (LAM) with tuberous sclerosis complex (TSC) is a difficult condition to manage. Video-assisted thoracoscopic surgery (VATS) may play a role in diagnosis and treatment of this situation. We present a case of right recurrent pneumothorax due to LAM with TSC in whom VATS was performed for pathological diagnosis and mechanical pleurodesis. The unique presentation of LAM in TSC was also discussed.

## Background

Pulmonary lymphangioleiomyomatosis (LAM) is usually detected in women of child-bearing age. It is characterized by the non-neoplastic proliferation of atypical smooth muscle cells within the lung parenchyma. Pulmonary LAM occurs in patients with tuberous sclerosis complex (TSC) with rate of 1.0 ~ 2.3% [1]. Recurrent pneumothorax in those patients is a challenging condition to manage. With the rapid advances of modern minimal invasive surgery, video-assisted thoracoscopic surgery (VATS) may play an important role in diagnosis and treatment for this condition. Here we describe the usage of VATS in a young woman with LAM and concomitant TSC, who suffered from right recurrent pneumothorax. VATS was performed successfully for pathological diagnosis of LAM, as well as definite treatment of pneumothorax. The unique presentation of LAM in TSC was also discussed.

## Case presentation

**Figure 1:**
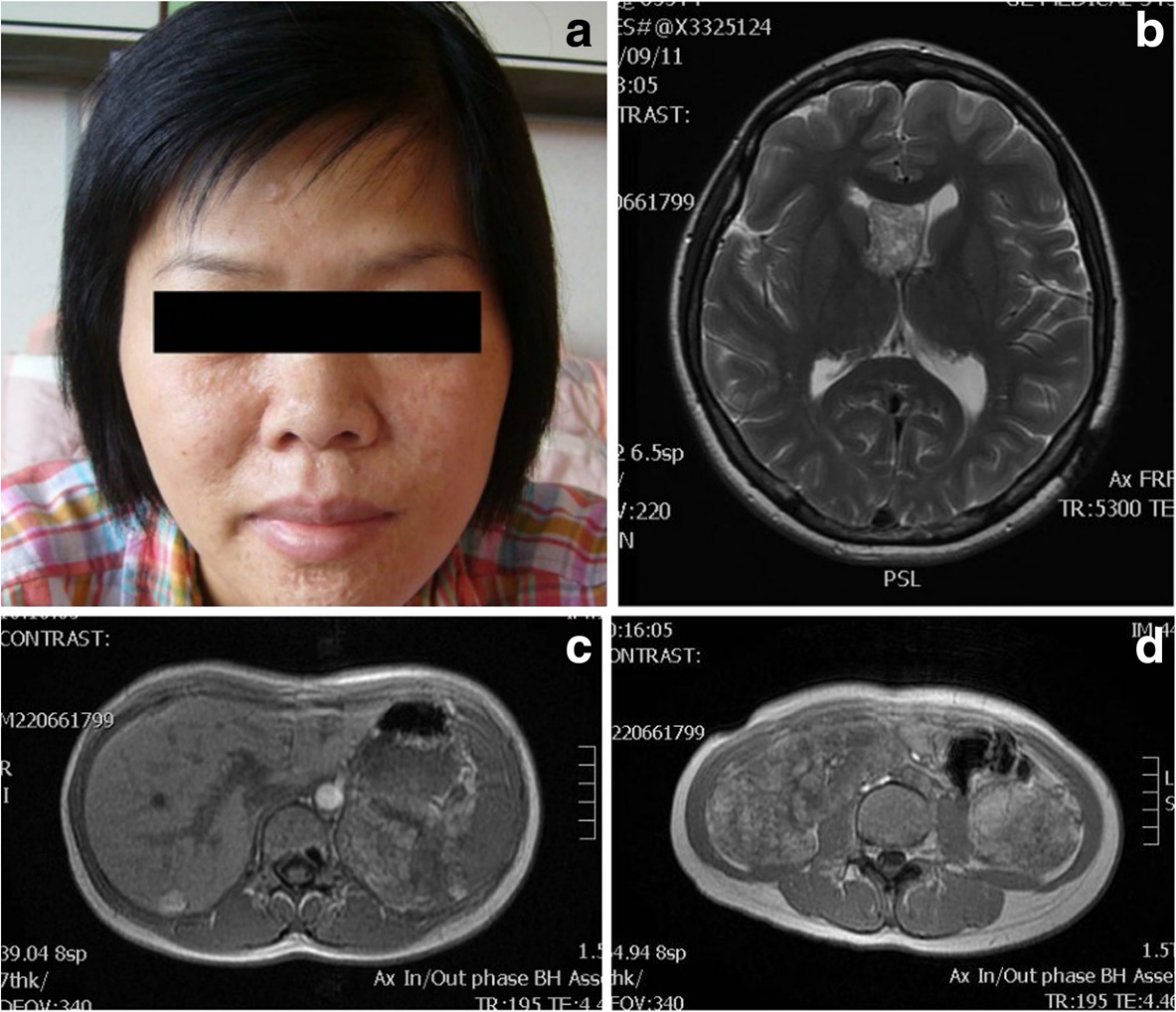
**Images of the patient. a**) sebaceous adenoma on the face, **b**) right ventricular subependymal giant cell astrocytoma, **c**) liver hamartoma and **d**) bilateral renal angiomyolipoma revealed by the magnetic resonance imaging (MRI).

**Figure 2:**
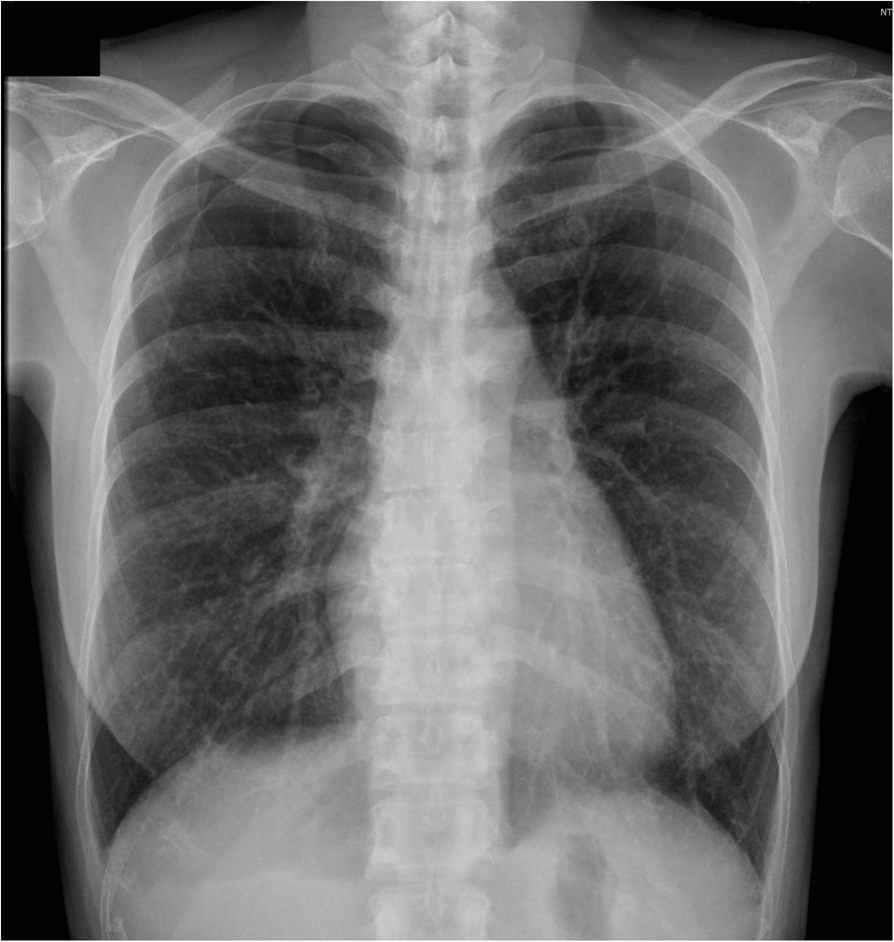
Chest plain film showed right pneumothorax with cystic change.

**Figure 3:**
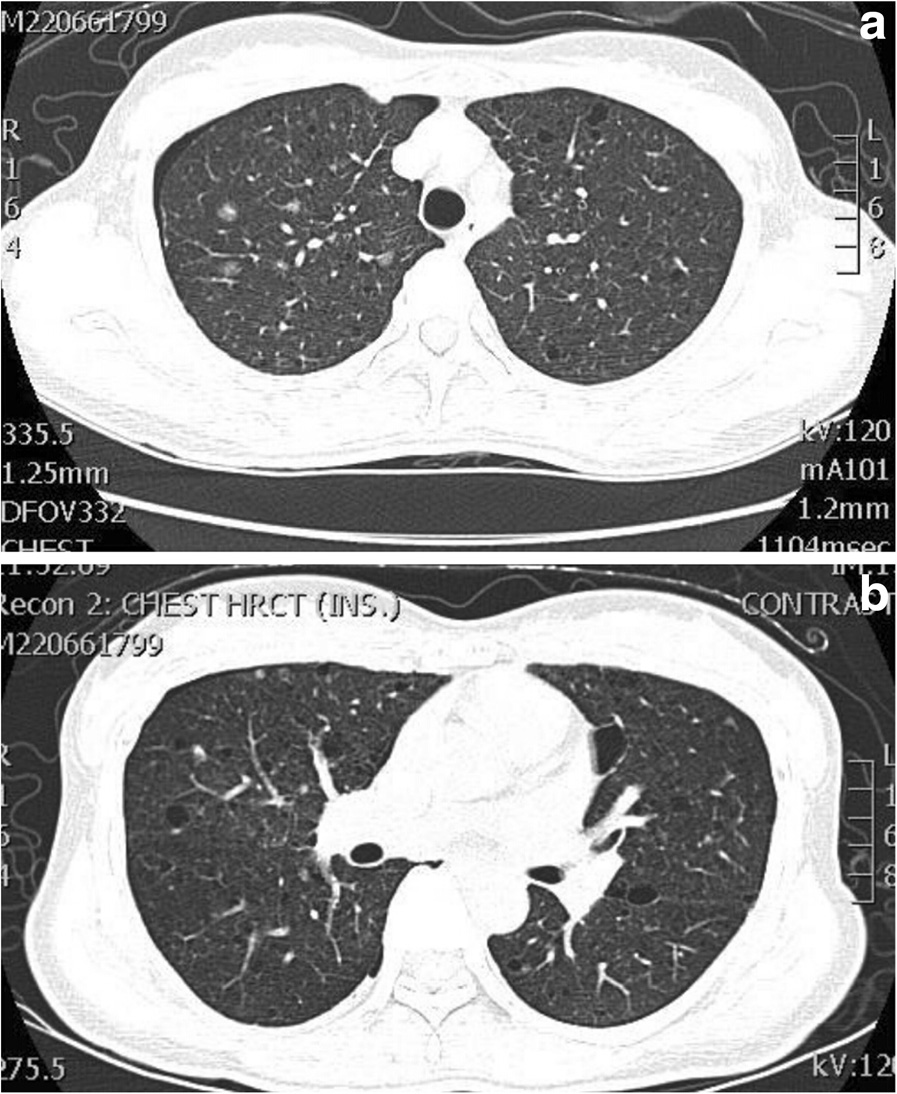
**a and b Chest HRCT.** HRCT showed bilateral numerous cystic lesions with right small pulmonary nodules.

**Figure 4:**
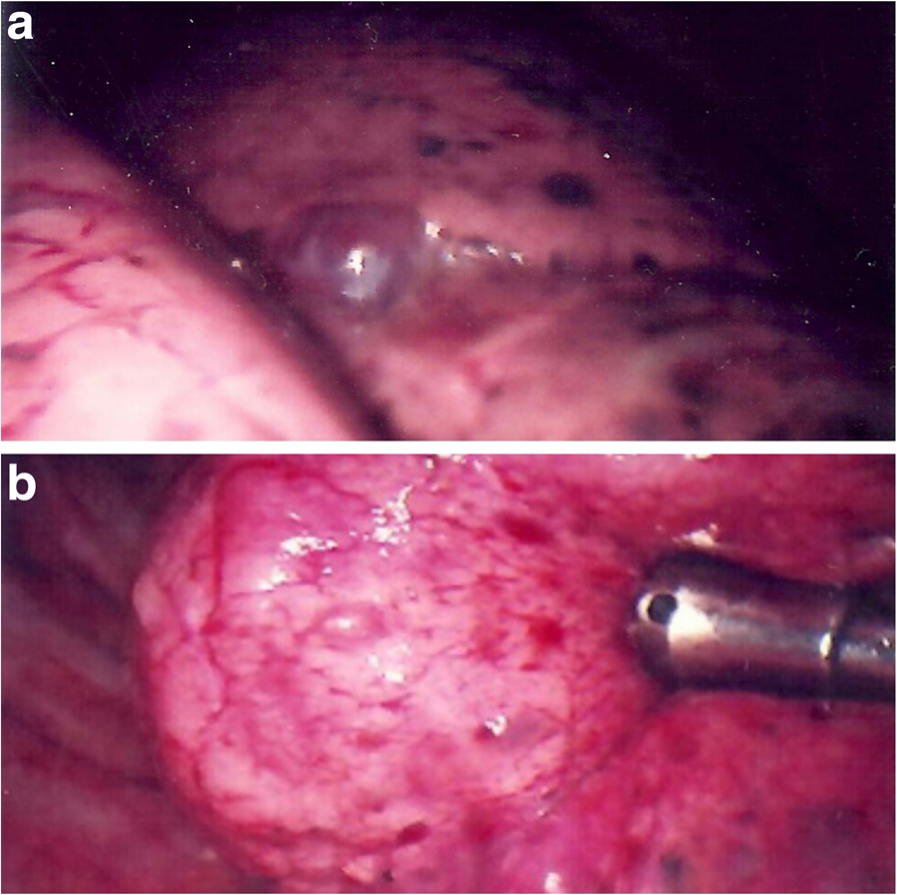
**a and b VATS pictures.** During VATS operation, a significant quantity of 2- to 3-mm diameter small cysts at the lung parenchyma and tonal change in the pleura were noted.

This 35 year-old Taiwanese female patient was diagnosed to have tuberous sclerosis complex (TSC) at the age of 30. She had been followed up at the outpatient department in our hospital because of a sebaceous adenoma on the face, right ventricular subependymal giant cell astrocytoma, liver hamartoma and bilateral renal angiomyolipoma (Figure 1). This time, she suffered from sudden onset of right chest pain with dyspnea for one day. She visited our emergent department for help where plain film showed right pneumothorax (Figure 2). High resolution computed tomography (HRCT) showed bilateral numerous cystic lesions with right small pulmonary nodules (Figure 3), compatible with presentation of LAM. Conservative treatment with oxygen therapy was conducted and she was discharged after pneumothorax improved. However, recurrent right pneumothorax occurred after one month. Therefore, we elected to perform VATS for treatment of pneumothorax and pathological diagnosis. During the operation, a significant quantity of 2- to 3-mm diameter small cysts at the lung parenchyma and tonal change in the pleura were detected (Figure 4). Right upper lobe lung wedge resection and apical mechanical pleurodesis were performed. After the surgery, no more air leakage was noted and chest plain film showed no pneumothorax. Pathologically, it revealed abnormal smooth muscle cell growth within the lung parenchyma. Furthermore, based on immunohistochemical staining, the cultured smooth muscle cells were found to be positive for human melanoma block (HMB)-45. Therefore, the diagnosis of LAM was confirmed. The post-operative course was smooth and she was discharged 4 days after the operation. Till now, she had been followed up in our outpatient department for 32 months without recurrent pneumothorax.

## Discussion

Tuberous sclerosis complex (TSC) is an autosomal dominant disorder with a birth incidence of around one in 10,000 [2] and a spontaneous mutation rate of ~ 65%. The main complex of symptoms of TSC are sebaceous adenomas on the face, renal angiomyolipoma, calcification of the ventricle wall, and subependymal giant cell astrocytoma with their probabilities reported to be 80%, 49%, 23%, and 6% respectively [1]. In comparison, for the LAM complex, it is as little as 1.0%–2.3% [1,3]. Lutembacher first described LAM in TSC in 1918, although he mistook the cystic and nodular changes for metastasis from renal fibrosarcoma. Dwyer described three cases of LAM in TSC and reviewed a further 31 cases [4], and Castro made a retrospective study of nine patients [3]. LAM, although rare, is an important cause of mortality in TSC. Shepherd et al. found lung disease to be the fourth most common cause of early mortality in TSC [5]. Average duration of survival from the time of diagnosis LAM was reported to be 4 ~ 8 years [4].

LAM predominantly affects females of childbearing age. The most common presenting symptoms are dyspnea (from pneumothorax and chylothorax), chronic cough, hemoptysis, wheeze and chest pain, but asymptomatic cases occur [4]. It can lead to cyanosis, respiratory failure and cor pulmonale. Pulmonary function tests show an obstructive more often than a restrictive pattern [3,4]. The histological images of LAM show aberrant growth of smooth muscle cells (LAM cells) around the alveolar walls, bronchi, lymph channels, and blood vessels. Immunohistochemically, LAM cells are positive for HMB-45 and progesterone receptor [6]. It was possible to diagnose LAM pathologically as well as immunohistochemically based on the lung specimen.

There is no consensus regarding the most sensitive diagnostic tests and the appropriate treatment for LAM. The intra-operative appearance of multiple and diffuse blebs over the entire surface of the lung, as illustrated in this case, was typical of this condition. Classically in LAM, CT scan shows bilateral thin-walled cysts distributed symmetrically throughout both lungs, however, unilateral lung involvement has been described. Confirmation of LAM requires histopathology analysis, which may be obtained by transbronchial, percutaneous or VATS biopsy. The risk of developing pneumothorax following transbronchial biopsy is between 1 and 6%, and it is even higher following percutaneous biopsy. VATS exerts its advantage on minimal surgical trauma as well as adequate specimen for pathological confirmation when bullectomy and pleurodesis or pleurectomy were performed [7,8]. Apical pleurodesis/pleurectomy was preferred both because it’s the routine in our institute and we anticipate the future need for lung transplantation. Talc pleurodesis was effective but not done in the female patient because of the young child-bearing age. Moreover, the risks of cancer (lung/ovarian), pregnancy, and future lung transplatation were high.

Various systemic therapeutic regimens have been reported, however, no one treatment offers a consistently effective response. Corticosteroids and cytotoxic agents have been used, but they seem to offer little benefit. Medical (progesterone, Tamoxifen, LHRH agonist) and surgical (oophorectomy, ovarian radiofrequency ablation) hormonal therapy have been used without consistent success. The long-term prognosis is often considered poor with many patients following a relentless deterioration after the onset of their symptoms. Lung transplantation can be an effective treatment for end-stage LAM; however, LAM cell migration into the transplanted lung can result in recurrence. A recent study also showed that sirolimus was an alternative drug for LAM [9].

## Conclusion

In summary, recurrent pneumothorax due to LAM is difficult to manage in patients with TSC. We report the experience of application of VATS in the condition, which exerted the advantages of minimal surgical trauma to treat and reduce pneumothorax recurrence, as well as to provide adequate lung tissue for diagnosis.

## Consent

Written informed consent was obtained from the patient for publication of this Case report and any accompanying images. A copy of the written consent is available for review by the Editor-in-Chief of this journal.

## Competing interests

The authors declare that they have no competing interests.

## Authors’ information

Chia-Fen Tsai and Chen-Hao Hsiao are co-first authors to this article.
